# Experimental study on the efficiency of dodecafluoro-2-methylpentan-3-one on suppressing lithium-ion battery fires

**DOI:** 10.1039/c8ra08908f

**Published:** 2018-12-18

**Authors:** Yujun Liu, Qiangling Duan, Jiajia Xu, Haodong Chen, Wei Lu, Qingsong Wang

**Affiliations:** State Key Laboratory of Fire Science, University of Science and Technology of China Hefei 230026 China; CAS Key Laboratory of Materials for Energy Conversion, University of Science and Technology of China Hefei 230026 P. R. China pinew@ustc.edu.cn

## Abstract

Currently, the effective and prompt suppression of lithium-ion battery fires is still challenging. Herein, a 38 A h prismatic ternary (Li(Ni_1/3_Co_1/3_Mn_1/3_)O_2_/graphite) battery with the size of 150 × 92 × 27 mm^3^ was adopted to investigate the suppression efficiency of dodecafluoro-2-methylpentan-3-one (C_6_F_12_O) in high capacity lithium-ion battery fires. Five doses of C_6_F_12_O agent including 0, 0.5, 1.0, 1.5 and 2.0 kg were adopted. It was concluded that as the dose of C_6_F_12_O agent increased, the peak temperature of the long surface and bottom of the cells first increased slowly and then decreased rapidly. The results indicated that the C_6_F_12_O agent first shows a negative inhibitory effect, which is then transformed into an inhibitory effect as the dose increases. This inhibitory effect grew distinct gradually with an increase in dose. It was found that in a 47.5 × 21.5 × 16 cm^3^ module box, the appropriate dose of C_6_F_12_O agent was 9.42 g W^−1^ h^−1^. Accordingly, these results have implications in the fire suppression design for lithium-ion batteries.

## Abstract 2

Currently, the effective and prompt suppression of lithium-ion battery fires is still challenging.

## Graphical abstract

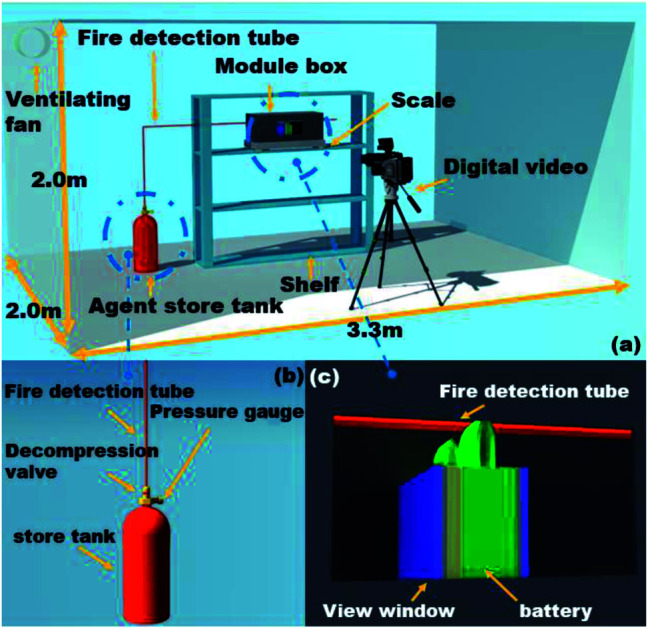

## 1. Introduction

Due to their advantages of high energy density, long lifespan, no memory effect and environmentally friendly nature, lithium-ion batteries have become the main medium for new energy storage systems. However, batteries may undergo thermal runaway^1^ under abuse conditions, including overcharging, overheating, and short circuiting, which may develop into violent burning and/or explosion without effective protective measures. Some lithium-ion battery fire accidents are summarized in Table 1.^2–4^ Thus, the issue of lithium-ion battery safety has attracted great concern.^4–8^

**Table 1:** Some fire accidents with lithium-ion batteries in recent years^2–4^

Date	Location	Accident	Possible reason
2016.01	Gjerstad, Norway	A Tesla Model S caught fire	Short circuit during charging
2016.07	Nanjing, China	An EV bus caught fire after a heavy rain	Short circuit
2017.03	Shanghai, China	A Tesla Model S caught fire	Unknown
2017.05	Beijing, China	Serial EV buses caught fire	External heat
2018.03	California, US	A Tesla Model X caught fire	Crash

Recently, many experimental and numerical investigations have been conducted with the aim to understand the thermal runaway and fire hazard of lithium-ion batteries, and some progress has been achieved. It was found that cells with an LiFePO_4_ (LFO) cathode seemed to show better safety characteristics, and batteries with a higher energy content performed the worst in safety tests.^5^ Thermal runaway is the most intractable safety issue for lithium ion batteries. When thermal runaway occurs, the temperature inside the battery reaches 870 °C,^6^ which is much higher than its surface temperature. Wang *et al.*^1^ and Feng *et al.*^4^ provided a comprehensive review on the thermal runaway mechanisms. Thermal runaway leads to a mechanism of chain reactions, during which the decomposition of the battery component materials occurs.^1,4^ Then, fires or explosions may occur after thermal runaway. Huang *et al.*^7^ investigated the combustion behavior of a lithium–titanate battery, and found that the fire hazard increased with the battery state-of-charge (SOC), and the battery combustion time became shorter with an increase in the SOC. Sun *et al.*^8^ conducted a toxicity analysis of the battery combustion products, which indicated that the SOC significantly affected the types of toxic combustion products, and 100% SOC even had the most serious toxicity.

Hence, aiming to reduce the thermal risk of lithium-ion batteries, many researchers^9–13^ have tried to achieve active protection by changing the internal structure of the battery. Nevertheless, existing technologies cannot fundamentally prevent thermal hazards of the battery, and fire accidents related to lithium-ion batteries still occur frequently. Consequently, in lithium ion battery-based energy storage systems, passive protection methods, such as extinguishing techniques, are important for the prevention and control of fire accidents at the present stage.

Many scholars and institutions conducted relevant experimental studies on suppressing lithium-ion battery fires.^14–22^ The fire test conducted by the National Technical Information Service (NTIS)^14,15^ showed that different Halon products could suppress battery fires, but the battery temperature would still increase after the flame was extinguished. Later, Egelhaaf *et al.*^16^ studied the suppression effect of a water agent with surfactant, a gelling agent and pure water agent on lithium ion battery fires. They proposed that water could be effective for lithium ion battery fires and additives helped to largely reduce the amount of water required for fire-fighting. Nevertheless, a lot white smoke was emitted after the fire was extinguishes. Then, a full-scale suppression test was conducted by the Fire Research Foundation.^17^ It was suggested that although battery fires could be quickly knocked down by a water jet flow within 25 s, the smoke and gas were still released after suppression. In the study of the Federal Aviation Administration (FAA),^18^ their results showed that water and other aqueous extinguishing agents such as water, AF-31, AF-21, Aqueous A-B-D, and Novec 1230 (C_6_F_12_O) were the most effective and the nonaqueous agents were the least effective. To find a high-efficiency extinguishing agent for lithium-ion battery fires, Wang *et al.*^19^ carried out a series of tests based on the lithium–titanate battery. Their results indicated that a single-cell or small-scale battery pack fire could be extinguished by heptafluoropropane. However, it was also found that the battery may reignite after it was put down due to the violent reactions inside the battery. In their other work,^20^ the extinguishing agents of CO_2_ and C_6_F_12_O were utilized to suppress lithium–titanate battery fires. Their results showed that C_6_F_12_O could suppress the fire within 30 s, whereas CO_2_ was incapable of fully extinguishing the flame over the full duration of the test. In the test of Det Norske Veritas and Germanischer Lloyd (DNV GL),^21^ F500, Fireice, PyroCool, aerosol and water were applied to test their extinguishing effects on battery fires. Their results showed that all the tested extinguishers could put down battery fires if they were used immediately upon the detection of a thermal spike. However, water was demonstrated to have the best ability to cool and maintain low temperatures in the battery. A water mist containing additives system was tested on an iron phosphate lithium ion battery fire.^22^ 5% F-500 solution and 5% self-made solution were verified to be more efficient than pure water in the water mist system.

To date, numerous experimental studies on lithium-ion battery fire suppression have been conducted. However, there are still many deficiencies in the current research. For example, fire extinguishing agents cause dramatic damage to batteries and modules, and the dose of agents may be hard to estimate during extinguishing.

Thus, as a new clean agent Halon alternative, C_6_F_12_O combines an outstanding extinguishing performance with an excellent environmental profile. In addition, the insulation and cooling performance of C_6_F_12_O are both outstanding, which is widely used in electrical fire protection. However, the application of the C_6_F_12_O agent in suppressing NCM lithium battery fires has not been reported to date. In this particular research, experiments were performed to investigate the inhibition efficiency of C_6_F_12_O on lithium-ion battery fires in a module box.

## 2. Experimental

### 2.1 Battery

A commercial ternary battery with a capacity of 38 A h and voltage of 4.2 V was used for the fire extinguishing experiments. The shape of the battery was prismatic, which was 150 mm, 92 mm and 27 mm in length, width and thickness, respectively. The cathode and anode electrode materials were Li(Ni_1/3_Co_1/3_Mn_1/3_)O_2_ (NCM) and graphite, respectively. Before the test, the batteries were charged to full state of charge (100% SOC) with its open circuit voltage of 4.2 V.

### 2.2 Experimental apparatus

**Fig. 1:**
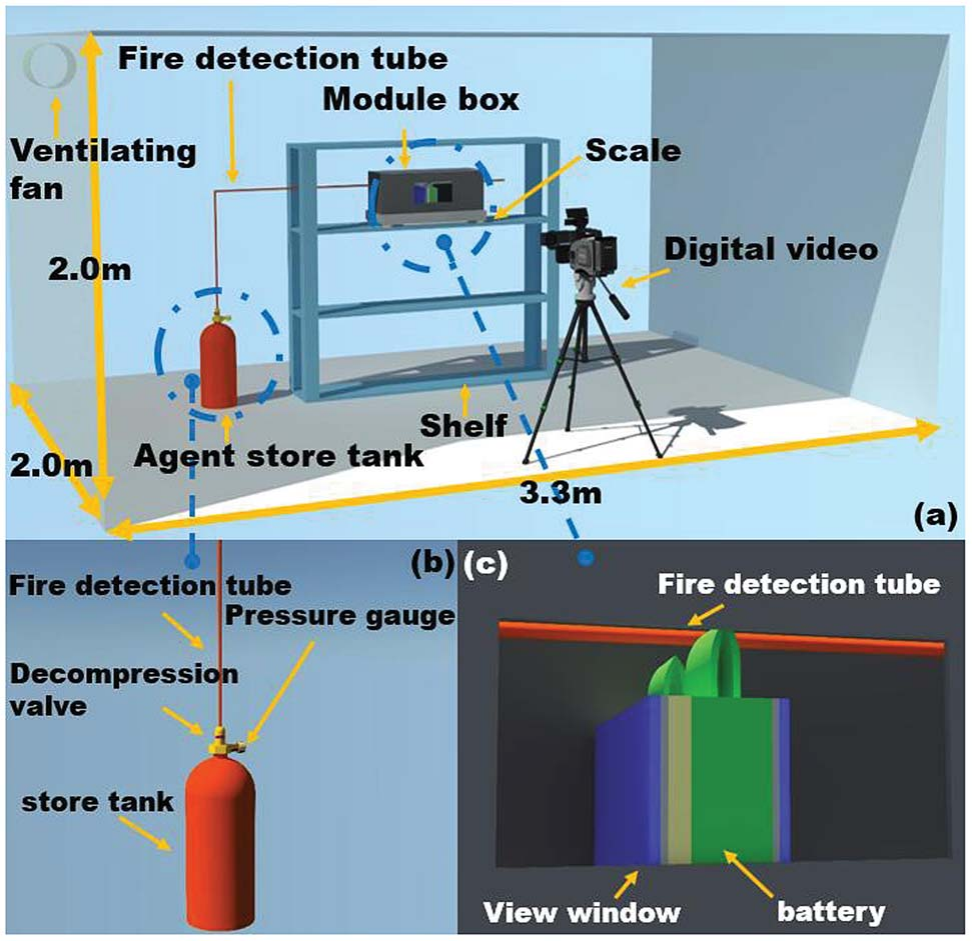
(a) Schematic view of the experimental apparatus, (b) details of the agent store tank and (c) details of the module tank.

A schematic view of the experimental platform is depicted in Fig. 1, which mainly consisted of an agent store tank, explosion-proof module box, fire detection tube, scale, temperature data acquisition system, several thermocouples and digital video. The size of the explosion-proof module box was 47.5 × 21.5 × 16 cm^3^, which was identical to the commercial single battery module. It was noted that once the battery underwent thermal runaway, it would generate significant amounts of smoke. For commodious observation, the view windows (10 × 5 cm^2^) were mounted on the side of the wall. A pressure relief vent was placed in the upper the box to emit smoke and reduce the internal pressure. The fire detection tube was placed above the cell safety valve with the height of 7.5 cm, and the tube was connected to the agent store tank, where the C_6_F_12_O and high-pressure N_2_ were stored. When the temperature in the protected enclosure rose to a critical threshold, the fire detection tube melted at the point of the highest affecting temperature. The C_6_F_12_O agent stored in the tube on the source of the fire was released through the melted hole of the tube.

**Fig. 2:**
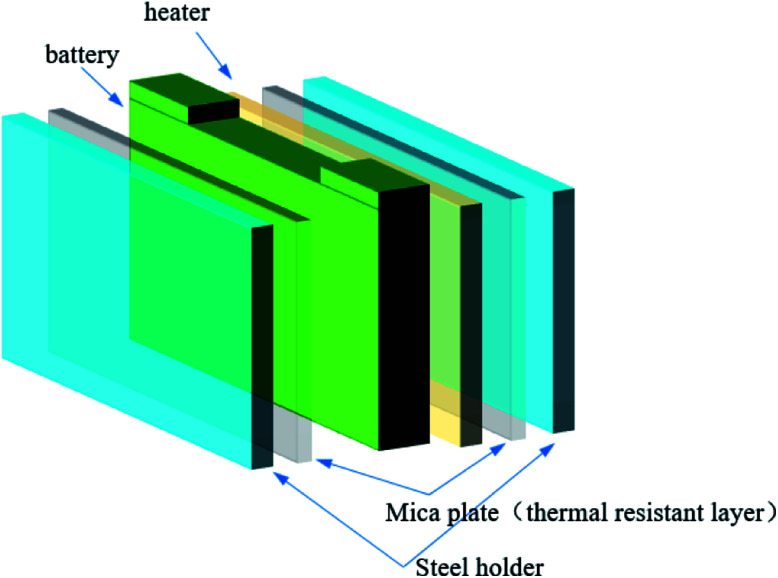
Placement of the battery, flaky heater, mica plate and steel holder.

Fig. 2 shows that a 400 W electric sheet heater with the same size as the battery was placed next to the battery to induce thermal runaway. The battery and the heater were trapped by two steel holders to simulate the close arrangement of the batteries. Two-mica plates were settled between the battery and the steel hold, and the heater and the steel hold, which simulated the real arrangement of the batteries in the module.

Different masses of C_6_F_12_O were packed into the agent store tank before the fire extinguishing test started. In the experiments, five experimental cases were conducted using 0, 0.5, 1.0, 1.5 and 2.0 kg C_6_F_12_O agent, which were initially filled into the tank. Then, nitrogen was pressed into the tank to let the interior pressure reach 2.5 MPa. The weight of the battery and agent store were measured before and after the experiment to determine the real mass loss of the battery and agent. Repeat tests were conducted in each condition to ensure the accuracy of the test. The specific experiment conditions are summarized in Table 2.

**Table 2:** Key parameters of the different experimental conditions

Item	Case 1	Case 1′	Case 2	Case 2′	Case 3	Case 3′	Case 4	Case 4′	Case 5	Case 5′
Loading dose (kg)	0	0	0.5	0.5	1.0	1.0	1.5	1.5	2.0	2.0
Pressure (MPa)	0	0	2.5	2.5	2.5	2.5	2.5	2.5	2.5	2.5
Ambient temperature (°C)	26.1	24.5	25.4	25.6	24.9	24.5	26.4	24.4	25.1	25.5
Release time (Xs after TR) (s)	—	—	3	5	3	3	3	3	5	3
Spraying time (s)	—	—	9	8	14	13	18	19	23	23
Battery mass loss (kg)	0.31	0.30	0.30	0.27	0.3	0.31	0.30	0.31	0.29	0.29
Used dose (kg)	0	0	0.36	0.36	0.86	0.86	1.39	1.38	1.89	1.86

During the test, the explosion-proof tank was settled on the scale, and the test was carried out in a confined compartment, as shown in Fig. 1. Once thermal runaway occurred, the heater was closed and the ventilating fan was opened.

### 2.3 Experimental condition settings and characteristic temperature

**Fig. 3:**
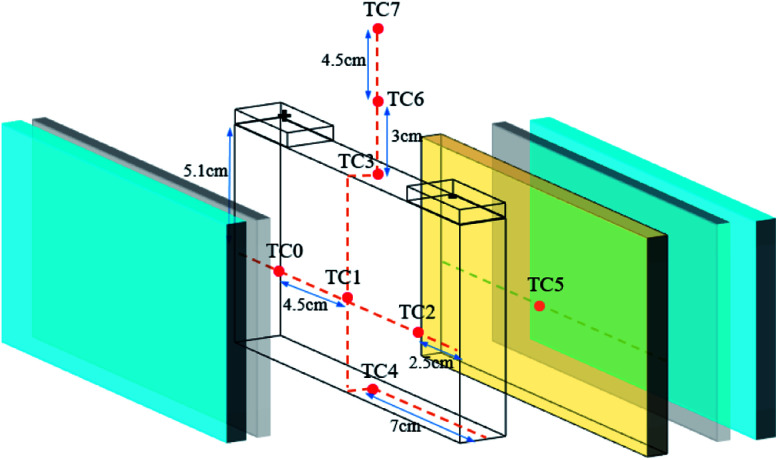
Arrangement of the thermocouples: one (TC5) on the surface of the heater, four (TC0–TC2, TC4) on the battery surface and three others (TC3, TC6, and TC7) above the safety valve.

Eight K-type thermocouples (TCs) were adopted to measure the battery surface and the flame temperatures. The positions of the TCs are shown in Fig. 3. The temperatures (*T*_lf_) in the long surface of the cell were monitored by TCs 0–2, while the temperature (*T*_uf_) in cell bottom surface was detected by TC4. A TC was always located on the surface of the heater element to verify adequate heat input. In addition, three TCs were placed 0, 30, and 75 mm above the safety valve to check the flame temperature during the thermal runaway and the extinguishing process.

**Fig. 4:**
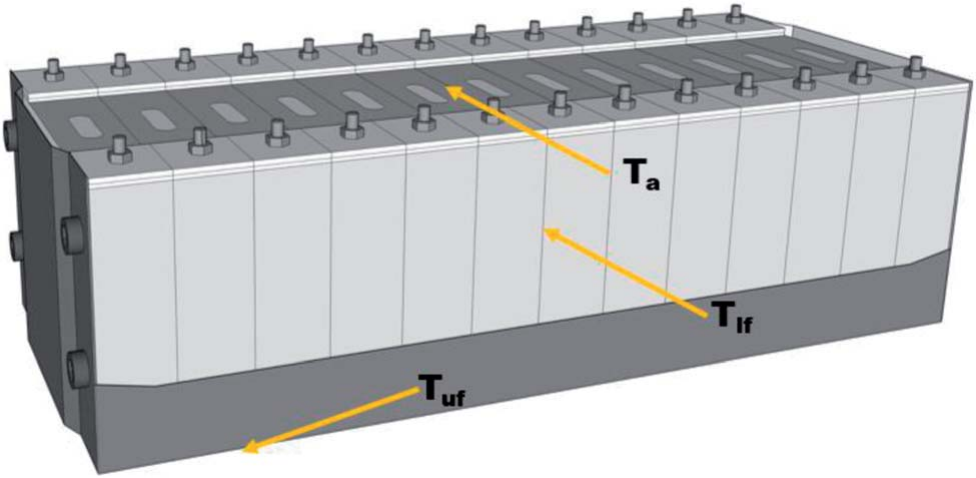
Schematic of the commercial battery module.

Fig. 4 shows a schematic diagram of the commercial battery module. When thermal runaway occurs, the heat transfer and thermal runaway propagation between adjacent batteries mainly depend on the heat conduction induced by the long surface. Similarly, the heat transfer between the batteries and the electronic circuit relies on the thermal radiation above the safety valve. Moreover, the heat transfer between the different modules mainly depends in the heat radiation spread by the bottom surface. Thus, to investigate the suppression and cooling effects of the C_6_F_12_O agent in different cases, the temperatures in the long surface (*T*_lf_), bottom surface (*T*_uf_), 7.5 cm above the safety valve (*T*_a_) and the mass loss during the suppression process in the different cases were compared.

## 3. Results and discussion

### 3.1 Processes of thermal runaway and extinguishing

**Fig. 5:**
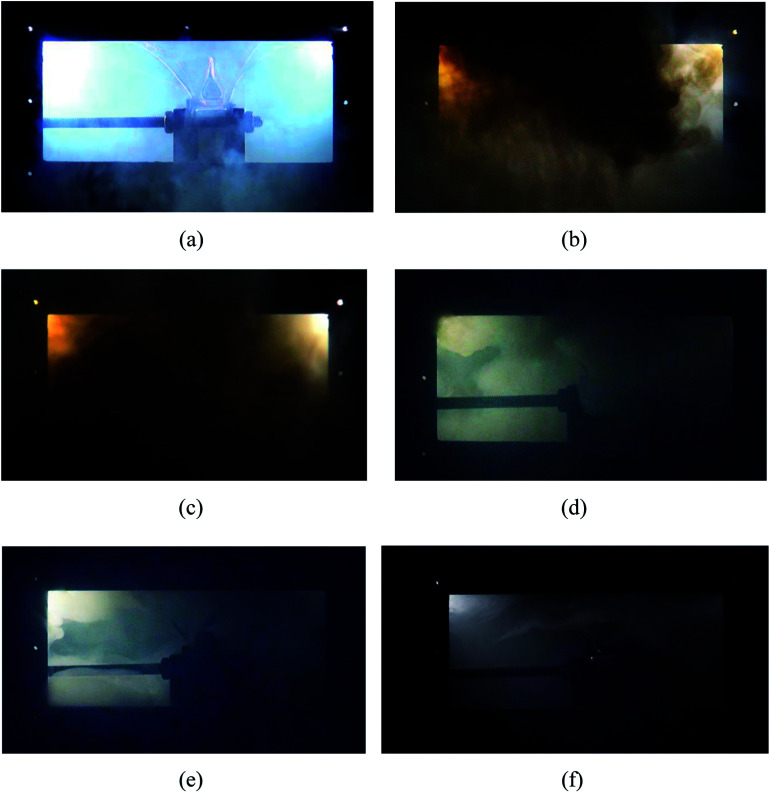
Extinguishing process in case 2. (a) After heating for 272 s, the safety value opened. (b) 1 s later, the dropped electrolyte was ignited, and black smoke poured out. (c) 3 s after the agent was applied. (d) The agent ran out and a significant amount of brown smoke was produced. (e) 60 s after the agent was applied, the fire was put out and the smog was diluted. (f) 120 s after the agent was applied, the smog almost vanished.

Fig. 5 shows the typical thermal runaway and fire suppression scenario in case 2. With the amount of heat accumulating (under heating process), various gases such as CO_2_ and H_2_ (ref. 23 and 24) expanded within the limited cell space, which caused the internal pressure to increase dramatically. Due to the restraint of the steel holders, deformation did not occur on the long surface, but it occurred to the side surface slightly. After heating for 272 s, as the cell reached the stress limit, the safety valve broke. White electrolyte together with some gas spilled from the safety valve in a remarkably short period of time, as shown in Fig. 5(a). 1 s later, with the ignition of the electrolyte and gas, the white smog turned black. Meanwhile, the anode and cathode materials were ejected together with the dense black smog. Due to the large amount smoke, the jet fire was not recorded by the digital camera. From Fig. 5(c), after the safety valve opened for 3 s, the fire detection tube melted due to the blistering hot gas and fire, and subsequently, the C_6_F_12_O agent was sprayed into the cell. Then 9 s later, the extinguishing agent release was completed, while the smog was still rather thick. As shown in Fig. 5(b)–(d), the black smog first turned brown then white. The initial black smoke was mainly composed of the ejected electrode materials and the incompletely combusted electrolyte. After the fire extinguishing agent was released, the combustion of the battery was chemically suppressed, and the combustion reaction was weakened, thereby leading to the black smoke turning brown gradually. Finally, due to the poor cooling effect of the agent, the electrolyte, which was not involved in the combustion reaction, was vaporized to white vapour at the high temperature. The final process took a long time of about 60 s. About 60 s after the agent was applied, the smog and vapour were diluted and the battery did not reignite.

The burning and suppression behaviors in the other cases were similar to that of case 2. Likewise, the cell fires in the other cases were put out and the cells did not reignite after the consumption of the agent. Due to the different rupture shapes of the safety valve, the timelines of the agent application may be diverse among the four cases. The experimental results show that the extinguishing agent seemed to mostly to be released within 3 to 5 s after the safety valve opened. It was also found that after the agent was applied for 60 s, the density of the smog and vapour was not reduced with an increase in the dose of the suppression agent.

**Fig. 6:**
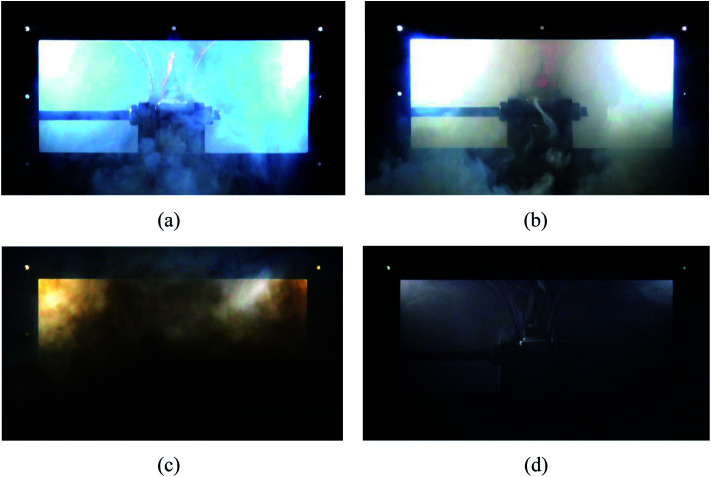
Extinguishing process in case 1, where no C_6_F_12_O was used. (a) After heating for 266 s, the safety value opened. (b) 3 s later, a jet fire formed above the safety valve. (c) Almost simultaneously, with the burning of the electrolyte and electrode materials, a large amount of brown smoke was released. (d) About 120 s later, the smog almost vanished.

Moreover, Fig. 6 shows the case where no C_6_F_12_O agent was used. Since no C_6_F_12_O used as an inhibitory agent, a jet fire was formed above the safety valve after the thermal runaway. Simultaneously, the duration of the brown smoke increased, which also indicates that the combustion reaction inside the battery in the case without any agent was more violent.

The results indicate that the efficiency of the C_6_F_12_O suppression agent was remarkable since it controlled the battery fire within 2 to 3 s and no obvious reignition appeared after the suppression. After the agent was applied, the battery produced a large amount of white smoke, which last for 60 s or even longer. The amount of white smoke was reduced with an increase in the dose of the agent, but the duration seemed to be independent of the dose of the agent.

### 3.2 Battery temperature response during thermal runaway and suppression process

**Fig. 7:**
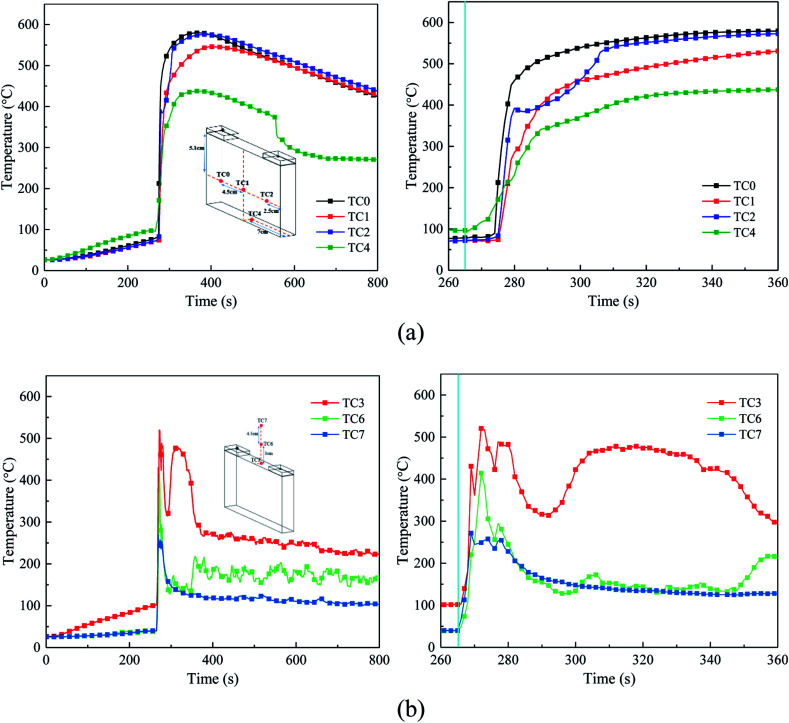
Temperature responses without extinguishing agent. (a) Temperature in the cell surfaces, where the right figure is a partial enlargement of the 260–360 s region. (b) Air and flame temperatures in above the safety valve, where the right figure is a partial enlargement of the 260–360 s region.

**Fig. 8:**
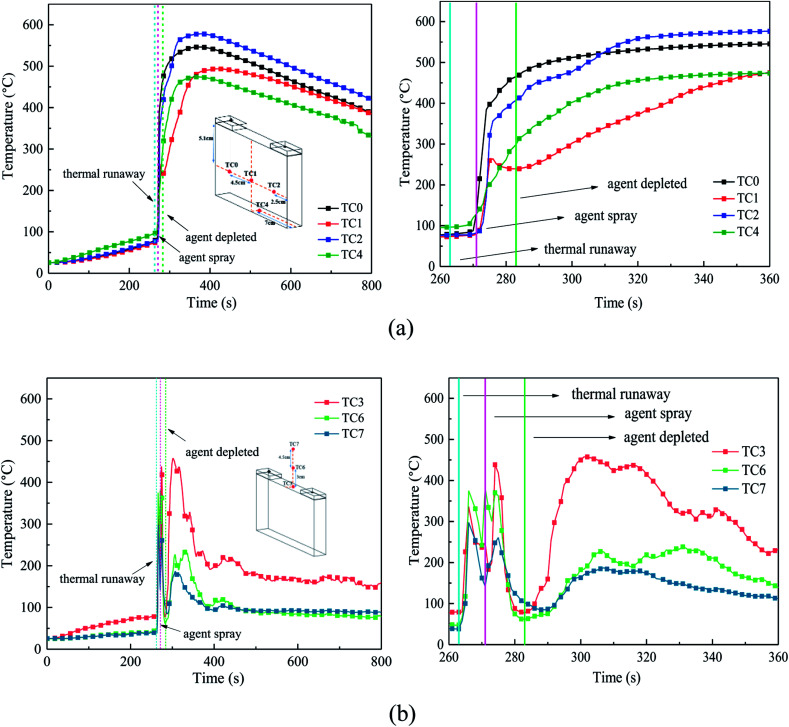
Temperature responses before and after the agent was applied. (a) Temperature in the cell surfaces, where the right figure is a partial enlargement of the 260–360 s region. (b) Air and flame temperatures above the safety valve, where the right figure is a partial enlargement of the 260–360 s region.

The temperature of the cell surface is the most persuasive parameter to indicate the characteristics of the thermal runaway and suppression process. Thus, four TCs were placed around the cell surface to measure the surface temperature, and three other TCs were arranged 0 cm, 3 cm, and 7.5 cm above the safety valve to gauge the air and flame temperature. Fig. 7 shows the temperature responses without agent in case 1, and Fig. 8 shows the temperature responses before and after the agent was applied in case 3.

From Fig. 7 and 8, the temperature of the cell increased dramatically with the thermal conduction and radiation from the heater. The increasing temperature promoted the decomposition of the solid electrolyte interface (SEI) film and the reaction between the electrolyte and anode.

After heating for nearly 240–265 s, thermal runaway occurred. A jet fire was formed at the safety valve, where the three TCs above the safety valve detected the high-temperature process. During the test, the maximum flame temperature of around 350–420 °C was much lower than the typical flame temperature, which may be a result of many uncontrollable factors such as agent stream pushing. About 9 s later, with the thermal runaway propagation inside the battery, the cell surface temperature increases dramatically from 80 °C to nearly 450 °C. Among the cases, the temperature rising rate (TRR) of the surface near the anode and cathode was the highest; whereas, the TRR of the bottom surface was much lower.

From Fig. 8(a), when the agent was completely released, the surface temperature still rose quickly, but the TRR decreased remarkably. This may be due to the following reasons: (1) the cell was clamped tightly by the holders, and the contact interface between the cell and agent was limited, thus the cooling efficiency of the agent was weakened and (2) although the flame and some of the reaction chains could be controlled and blocked by the C_6_F_12_O agent, it was nearly impossible to hinder all the violent reactions inside the battery. Thus, the battery surface temperature still increased, but the TRR was much slower than before. Notably, there was a minor temperature decline in the center of the cell long surface when the safety valve was opened, which is attributed to the ejection of the active substance and the cooling process of high-pressure stream inside the battery.

From Fig. 7(a) and 8(a), in case 1 without C_6_F_12_O agent, the average TRR of the cell surface was 4.0175 °C s^−1^, while in case 3 it was 3.795 °C s^−1^, which means that the C_6_F_12_O agent removed some of heat and delayed the propagation of heat.

After the C_6_F_12_O agent finished, the surface temperatures were vastly different in the different locations of the cell. It was found from Fig. 8(a) that the peak temperature at the bottom and the center of the long surface was about 470 °C and 490 °C, while that at the long surface near the anode and cathode as almost 570 °C and 550 °C, respectively. Simultaneously, the temperature above the safety valve decreased gradually, then fluctuated around an average value, which decreased from the surface of the safety valve to the upper air. The average value at the surface of the safety valve was nearly 180 °C, while the temperatures at 3 and 7.5 cm above the safety valve were all almost 90 °C.

In summary, the experimental results indicate that the C_6_F_12_O agent cannot reduce the battery temperature immediately after the extinguishing process. When the C_6_F_12_O agent finished, the battery temperature still increased. However, when the dose of C_6_F_12_O agent was different, the peak temperature of each surface of the battery was different, which would be discussed in the next section.

### 3.3 Suppression efficiency of C_6_F_12_O

**Fig. 9:**
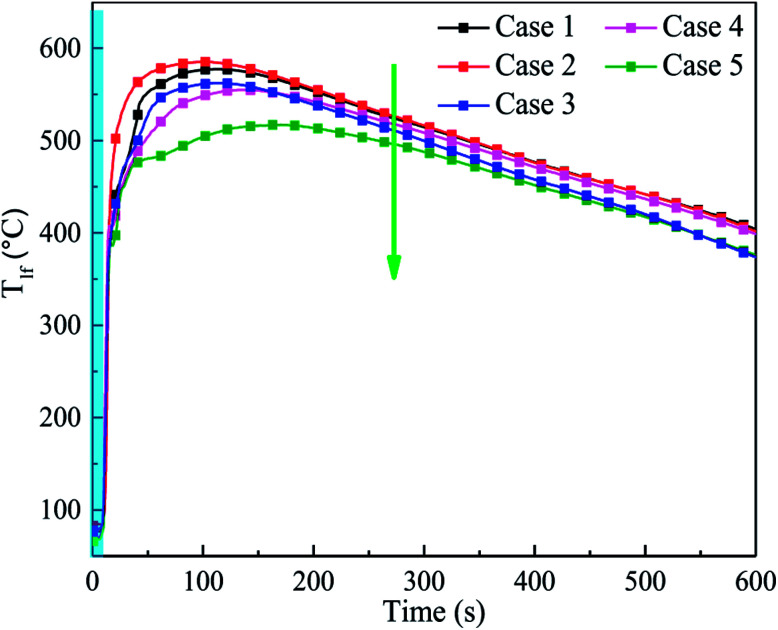
Temperature responses of the cell long surfaces after the agent was applied in cases 1–5.

To study the suppression efficiency of C_6_F_12_O with different doses, the characteristic temperature responses and the mass change were compared. Fig. 9 shows the temperature responses of the cell long surfaces after the agent was applied. The blue band in Fig. 9 is used to represent the release time of the C_6_F_12_O agent. From Fig. 9, the peak value of *T*_lf_ significantly decreased as the amount of agent increased. The average TRR in cases 2–5 from applying agent to reaching the peak temperature was 5.5, 4.08, 3.7 and 2.7 °C s^−1^, respectively. The results suggest that the exothermic reaction inside the battery becomes much more moderate with an increase in the amount of C_6_F_12_O agent, *i.e.* as the dose increases, the cooling effect of the agent becomes much more pronounced.

It was also found that the TRR and peak temperature in case 1 were lower than that in case 2, as shown in Fig. 9. This is mainly because a small amount of agent may promote a temperature increase in the cell, which indicates the peculiar performance of the C_6_F_12_O in extinguishing battery fires.

**Fig. 10:**
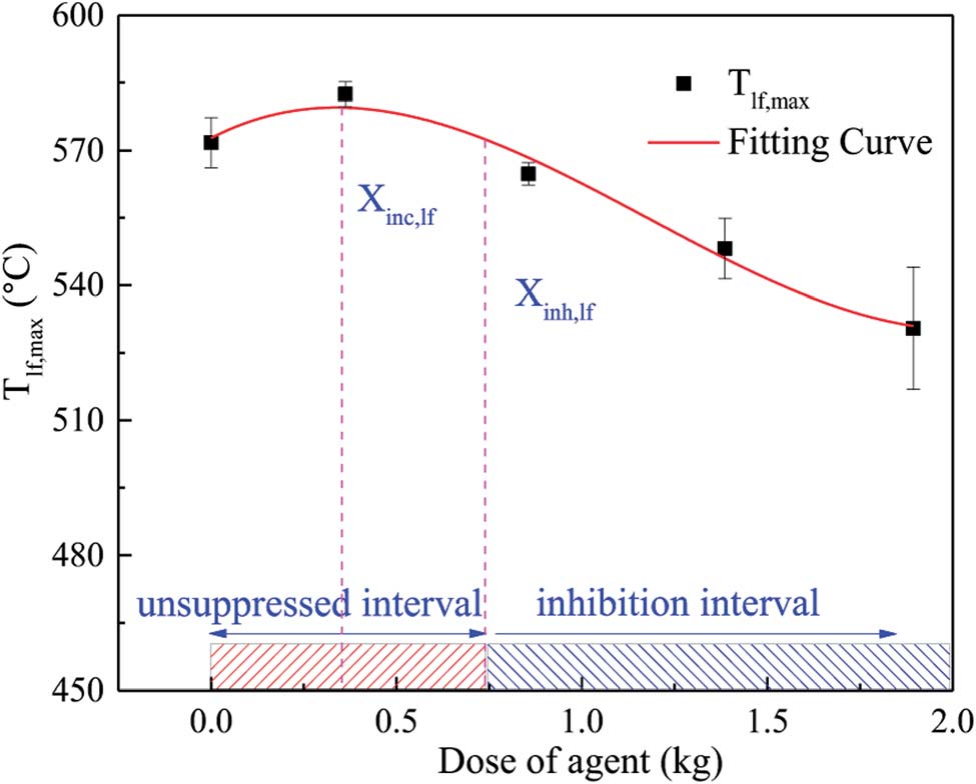
Fitting curve of *T*_lf,max_ and agent dose in cases 1–5.

The relationship between the peaking of *T*_lf_ (*T*_lf,max_) and the dose of agent (*X*_in_) is shown in Fig. 10, which was fitted as a third-order polynomial curve. The *T*_lf,max_ in each case was denoted by the average value of several repeated tests. According to Fig. 10, the curve could be segmented into 2 characteristic regions. In the first region, as *X*_in_ increased, *T*_lf,max_ increased slightly, then peaked at the critical dose (*X*_inc_). Thereafter, in the second region, for inhibitor loadings greater than *X*_inc_, *T*_lf,max_ decreased gradually with an increase in *X*_in_. In the system, there was an unsuppressed interval and inhibition interval, which depend on the dose of the C_6_F_12_O agent. When the dose of C_6_F_12_O agent exceeded the inhibition critical dose (*X*_inh_), the C_6_F_12_O agent played an inhibition role; otherwise, the agent exhibited a negative effect on the inhibition.

This peculiar phenomenon may be related to the special nature of C_6_F_12_O. In a rich-burn system, the inhibition effect becomes more obvious as the dose increasing.^25^ However, in our experiments, the batteries were ignited in a semi-closed tank, in which oxygen was amply furnished. Thus, the battery fire inside the tank is deemed as lean combustion. In the lean-burn system, when the amount of fire extinguishing agent was limited, the amount of fluorine atoms is less than hydrogen atoms after the release of C_6_F_12_O. There is enough H atoms to form HF, which is the most stable product of fluorine, and more heat is released in this process compared to the formation of other fluorine species. At *X*_inc_, the fluorine to hydrogen ([F]/[H]) atomic ratio is 1,^25^ thus, *T*_lf,max_ reached the peak value under all the conditions. In the second region, *T*_lf,max_ decreased gradually. This is because above *X*_inc_, there is insufficient H atoms in the system to form HF, and instead partially oxidized species (such as COF_2_ and CF_4_) are formed, leading to less heat release. Another theory indicates^26^ that at low inhibitor loadings and over-ventilated conditions, adding agent made the system more reactive, while at higher loadings, higher concentrations had little suppression effect on the reactivity.

**Fig. 11:**
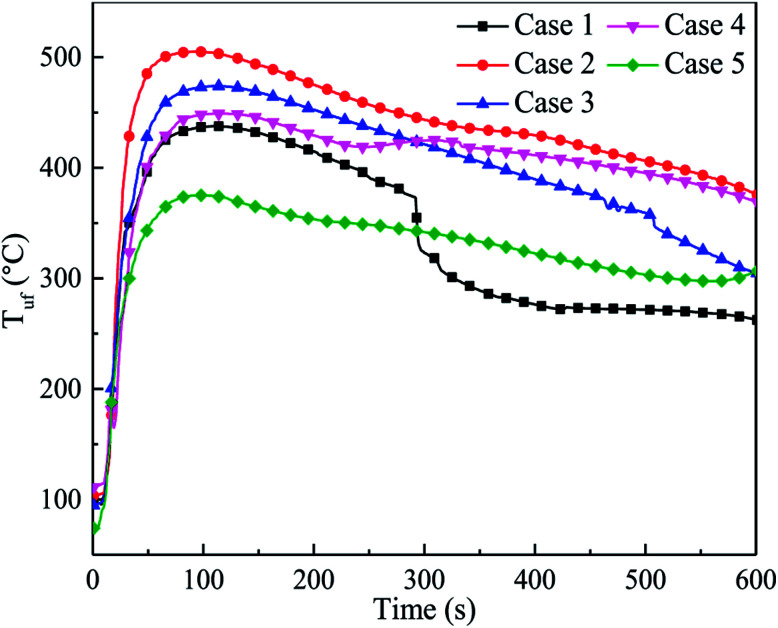
Temperature responses of the bottom surfaces of the after the agent was applied in cases 1–5.

However, due to the uneven distribution of the agent, the inhibition effect of C_6_F_12_O on different positions in the cell may be dramatically different. Fig. 11 shows the temperature responses of the bottom surfaces of the cells after the agent was applied in cases 1–5. Both the average TRR and the peak temperature for case 2 and case 3 were significantly higher than that in case 1. The TRR and *T*_lf,max_ in case 4 slightly increased compared to case 1, which illustrates that C_6_F_12_O in case 4 still has an adverse effect in inhibiting the temperature increase on the bottom surface.

**Fig. 12:**
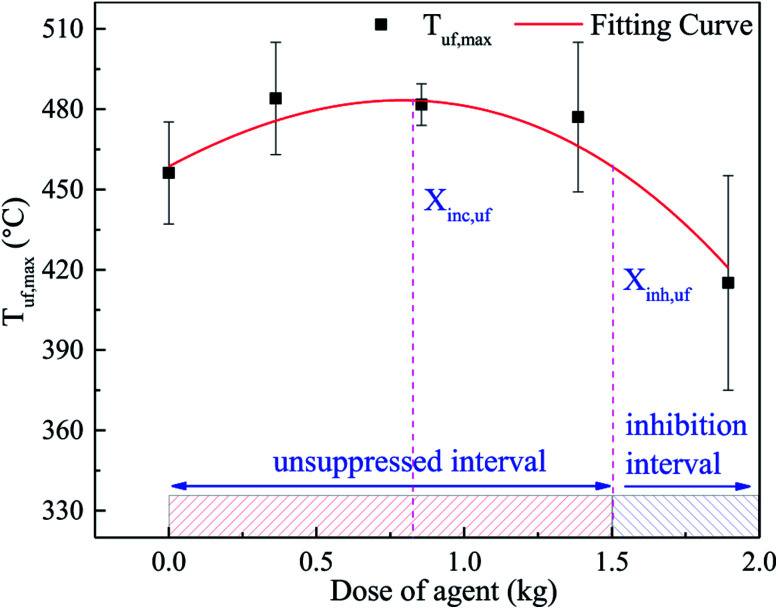
Fitting curve of *T*_uf,max_ and agent dose in cases 1–5.

The *T*_uf,max_ was fitted in a third-order polynomial curve as well, as shown in Fig. 12. It was found that the trend of the curve was almost the same as that in Fig. 10. Nonetheless, due to the uneven distribution of the agent, the critical dose of the different positions was different. Compared to the long surface, the critical dose (*X*_inc,uf_) and the unsuppressed interval in the bottom surface seemed lager. When thermal runaway arose, plenty black smoke was produced, which contained numerous unreacted electrode materials, including graphite. Thus, a large amount of graphite dust was suspended in the fundus of the explosion-proof tank due to its larger relative molecular mass. As a result, the agent concentration at the fundus was much lower than that at the long surface. Hence, *X*_inc,uf_ was larger than *X*_inc,lf_, and the unsuppressed interval was much more extensive.

**Fig. 13:**
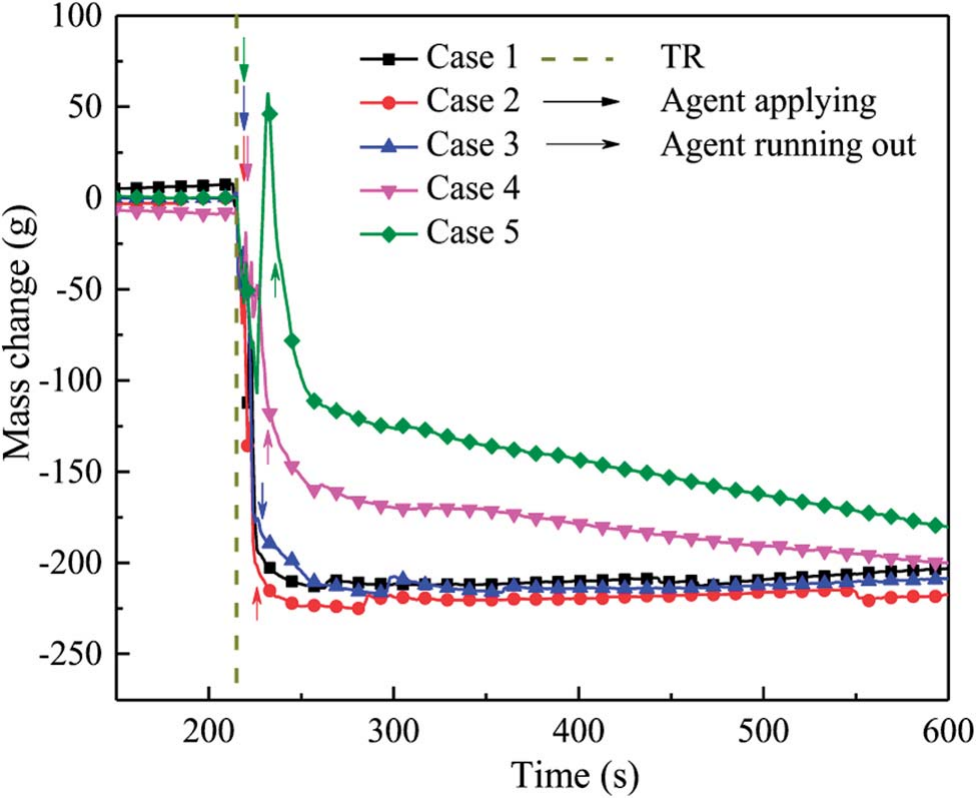
Mass change in cells after the agent was applied in cases 1–5.

During the experiment, the mass change in the experimental system was also determined, as shown in Fig. 13. When thermal runaway occurred in the cell, the quality of the system decreased rapidly due to the release of the electrolyte and electrode material. Although the was agent applied, the mass of the system still decreased. This is mainly because the C_6_F_12_O could not be spread into the interior of the cell, where the violent reaction was continuing, and the material was quickly released.

When the suppression effect improved, the system residual quality (*Q*_sr_) was much higher, for the decomposition, which led to the mass loss being weakened by C_6_F_12_O. From Fig. 13, when the extinguishing agent was finished, *Q*_sr_ in case 2 was lower than that in case 1. The *Q*_sr_ in case 3 was slightly higher than case 1, which indicates that a small amount of agent exerts a negative effect on the inhibition. The *Q*_sr_ in case 4 and case 5 was higher than that for the other cases, but the system quality still declined after the agent was released. This implies that the combustion reaction inside the cell was still taking place; however, the reaction rate and material consumption were both at a low level. The *Q*_sr_ in case 4 and case 5 was higher, which is possibly because the amount of F atoms is greater than H atoms in the system after the agent was released, and then some fluorine species substances (CF_4_, *etc.*) with a larger molecular weight were generated and deposited in the bottom part of the module box, which increased the quality of the system. Thereafter, the quality of the system decreased slowly with the diffusion of gases and deferred reaction inside the battery. For case 2 and 3, which applied less C_6_F_12_O, the amount of H atoms was sufficient to consume all the F atoms to generate HF. However, the molecular weight of HF is lower than air, thus HF was released from the top pressure relief hole in the module box during the test. In addition, when the dose of agent was limited, the inhibitory effect was much poor, thus the reaction inside the cell was more severe and the *Q*_sr_ was much lower. For the system quality in cases 1, 2 and 3, the slight increasing process may be responsible for the deposition of suspended graphite powder in the module box.

In summary, as the dose of C_6_F_12_O agent increased, the residual quality of the battery remined higher and the mass change became much slower, which indicate that a larger amount of agent can slow down the reaction, but it may not prevent the reaction. Moreover, more C_6_F_12_O cannot fundamentally interrupt the reaction, but only delay the reaction process, which can provide more time for system alert and personnel evacuation.

### 3.4 Proper choice of C_6_F_12_O dose

For a lithium-ion battery system, the combustion type of this system should be first defined. If the combustion is a lean-burn process, the critical inhibition dose needs to be considered. However, if the combustion is a rich-burn process, the critical inhibition dose may not need to be considered because the inhibition effect becomes better with an increase in the dose of agent.^26^ According to the above analysis, due to the uneven distribution of the agent, the critical inhibition dose in the different parts of the battery pack may be significantly different, as shown in Fig. 10 and 12. Specifically, for a certain lithium-ion battery system, the proper dose of C_6_F_12_O may be determined through the coupling of several characteristic surface inhibition critical doses. The proper suppression dose of a single cell fire in the 47.5 × 21.5 × 16 cm^3^ module box should be more than 1.504 kg, as calculated by this method. Thus, in the other similar lithium-ion battery-based systems, the proper dose of C_6_F_12_O agent is 9.42 g W^−1^ h^−1^. However, the final dose should be evaluated by combining the weight, cost and other comprehensive factors since only the inhibitory effect is considered for this method.

## 4. Conclusions

In this work, the efficiency of C_6_F_12_O on suppressing the lithium-ion battery fires was experimentally investigated. The primary results are as follows:

(1) The present results show that an open fire can be extinguished by C_6_F_12_O within 2 to 3 s. The amount of the smoke released during thermal runaway will be reduced with an increase in the dose of C_6_F_12_O, while the duration has nothing to do with the dose. Moreover, when the dose of agent is limited, the battery may undergo reignition due to the deep smoldering inside the prismatic battery.

(2) In the case with steel holders, the cooling effect of C_6_F_12_O is unobvious. Therefore, to control the battery temperature immediately after fire extinguishing, other auxiliary means such as liquid cooling are required.

(3) It was found that the relationship between the dose of the agent and inhibitory effect is not a simple linear relationship. With an increase in the dose, the C_6_F_12_O agent first exerts a negative effect on the inhibition, and then exhibits an inhibitory effect. For doses larger than the critical value (*X*_inc_), the inhibitory effect becomes better. A critical inhibition dose exists in the system, but due to the uneven distribution of the agent, the critical inhibition dose varies with different locations in the battery. In this research, after using C_6_F_12_O, the peak temperature of the long surface with 0, 0.5, 1.0, 1.5 and 2.0 kg C_6_F_12_O was 571.8 °C, 582.7 °C, 564.4 °C, 547.9 °C and 530.2 °C and the peak temperature of the bottom was 456.1 °C, 483.8 °C, 481.4 °C, 476.7 °C and 415.7 °C, respectively. Thus, the proper dose of C_6_F_12_O may be determined through the coupling of several characteristic surface inhibition critical doses. In the experimental module box, the proper dose of the C_6_F_12_O agent is 9.42 g W^−1^ h^−1^.

## Conflicts of interest

There are no conflicts to declare.

## Supplementary Material
